# Integrating Differential Evolution Optimization to Cognitive Diagnostic Model Estimation

**DOI:** 10.3389/fpsyg.2018.02142

**Published:** 2018-11-06

**Authors:** Zhehan Jiang, Wenchao Ma

**Affiliations:** ^1^Department of University Libraries, University of Alabama, Tuscaloosa, AL, United States; ^2^Department of Educational Studies, University of Alabama, Tuscaloosa, AL, United States

**Keywords:** differential evolution optimization, cognitive diagnostic model, LCDM, estimation, EM algorithm

## Abstract

A log-linear cognitive diagnostic model (LCDM) is estimated via a global optimization approach- differential evolution optimization (DEoptim), which can be used when the traditional expectation maximization (EM) fails. The application of the DEoptim to LCDM estimation is introduced, explicated, and evaluated via a Monte Carlo simulation study in this article. The aim of this study is to fill the gap between the field of psychometric modeling and modern machine learning estimation techniques and provide an alternative solution in the model estimation.

Assessments have been widely used in education as a part of a summative program for many purposes, such as evaluating whether students have reached the desired proficiency level and determining whether students should be given a scholarship. However, in the past decades, stakeholders have shown a strong interest in the information of students' strengths and weaknesses of their knowledge and skills. This has led to fruitful exploration in the field of psychometrics of how to extract diagnostic information to enhance classroom instruction and learning. Cognitive diagnostic models (CDMs) are a set of psychometric models developed to identify whether a student masters a set of fine-grained skills, such as addition, subtraction, multiplication, and division in math ability assessments. For example, question “2+4–1” measures addition and subtraction, and “4 × 2/3” measures multiplication and division. Although it seems straightforward to conclude that a student may not master addition or subtraction if s\he fails 2+4–1, it is indeed much more complicated in practice in that students may answer a question correctly by guessing or fail a question due to carelessness. As a result, formal psychometric models such as CDMs should be employed for data analysis to make sure the inferences are valid. In addition to educational testing, CDMs are useful in psychological measurement. For example, the literature indicates that neuro-vegetative symptoms are a general construct that contains three attributes: depression (DEP), fatigue (FAT), and sleeplessness (SLE; Rabinowitz et al., 2011). Using CDMs allows researchers/practitioners to investigate the attributes of a given patient. Among the item data types, a binary scale is the most common one that has been adopted in many surveys and measures.

Prior to the data analysis using CDMs, whether a skill is required for answering a question needs to be determined by content experts and/or cognitive psychologists and specified in a binary matrix (Q-matrix; Tatsuoka, 1983) as illustrated in Table 1 such that theory-granted structure can be applied to the measurement of interest. Rows of the Q-matrix represent questions and columns represent skills. Element 1 indicates that the skill is measured by the question and 0 indicates that the sill is not measured.

**Table 1:** A Q-matrix sample.

	**Skill 1**	**Skill 2**	**Skill 3**
Question 1	1	0	0
Question 2	1	0	1
Question 3	0	1	1
Question 4	1	1	0

Recent advances in modeling development have produced several general CDMs, such as the Log-linear CDM (LCDM; Henson et al., 2009) and, equivalently, the generalized Deterministic Input; Noisy “And” gate model (G-DINA; de la Torre, 2011). The LCDM provides great flexibility such as (1) subsuming most latent variables, (2) enabling both additive and non-additive relationships between skills and questions simultaneously, and (3) syncing with other psychometric models. Rupp et al. (2010, p. 163) proved that LCDM can be constrained to core CDMs such as Deterministic Input; Noisy “And” gate (DINA; Junker and Sijtsma, 2001) model, Noisy Input; Deterministic “And” gate (NIDA; Junker and Sijtsma, 2001) model, and the Reduced Reparameterized Unified Model (RRUM; Hartz, 2002), and Deterministic Input; Noisy “Or” gate (DINO, Templin and Henson, 2006) model.

The LCDM is essentially a restricted latent class model (Day, 1969; Wolfe, 1970; Titterington et al., 1985), and mathematically, it can be defined as:

(1) $$P\left(Y_{p}=y_{p}\right)=\sum_{c=1}^{C}\left(v_{c}\prod_{i=1}^{I}\pi_{ci}^{y_{pi}}{\left(1-\pi_{ci}\right)}^{1-y_{pi}}\right),$$

where ***y***_*p*_ = (*y*_*p*1_, *y*_*p*2_, …, *y*_*pI*_) is the binary response vector of person *p* on a test comprised of *I* items, and element *y*_*pi*_ is the response on item *i*. *v*_*c*_ is the probability of membership in latent class *c*, and π_*ci*_ is the probability of correct response to item *i* by person *p* from latent class *c*. The log-likelihood of observing item responses of *N* persons can be expressed as

(2) $$L=\sum_{p=1}^{N}\log\left\{\sum_{c=1}^{C}\left(v_{c}\prod_{i=1}^{I}\pi_{ci}^{y_{pi}}{\left(1-\pi_{ci}\right)}^{1-y_{pi}}\right)\right\}.$$

Further, Equation 2 can also be converted to:

(3) $$L=\sum_{p=1}^{N}\log\left\{\sum_{c=1}^{C}\left(\exp\left(\log(v_{c})+\log(\prod_{i=1}^{I}\pi_{ci}^{y_{pi}}{\left(1-\pi_{ci}\right)}^{1-y_{pi}})\right)\right)\right\},$$

where $\log\left(\prod_{i=1}^{I}\pi_{ci}^{y_{pi}}{\left({1-\pi }_{ci}\right)}^{1-y_{pi}}\right)$ can be replaced by $\overset{I}{\underset{i=1}{\sum }}\log\left(\pi_{ci}^{y_{pi}}{\left({1-\pi }_{ci}\right)}^{1-y_{pi}}\right)$ due to the mathematical property of *log* operation.

Assume the number of attributes is *A*, the mastery profile of the attributes for a random person is denoted by **α** = (α_1_, α_2_, …, α_*A*_), where element α_*a*_ is either 1 or 0. In total, there are 2^*A*^ possible attribute profiles and correspondingly 2^*A*^ latent classes. For example, when *A* = 4, a person with attribute profile **α** = (1, 1, 1, 0) has mastered the first three attributes except the last one. As illustrated in Table 1, a Q-matrix of size I^*^A is necessary for a LCDM, where the (*i, a*) element *q*_*ia*_ is 1 when item *i* measures attribute *a* and 0 otherwise. The conditional probability of person *p* with attribute profile **α**_*c*_ answering item *i* correctly can be written by

(4) $$\pi_{ci}=P(y_{pi}=1|\alpha_{c})=\frac{\exp\left(\lambda_{i,0}+\lambda_{i}^{T}h\left(\alpha_{c},q_{i}\right)\right)}{1+\exp\left(\lambda_{i,0}+\lambda_{i}^{T}h\left(\alpha_{c},q_{i}\right)\right)},$$

where ***q***_*i*_ is the set of Q-matrix entries for item *i*, λ_*i*, 0_ is the intercept parameter, where **λ**_*i*_ represents a vector of length 2^*A*^ − 1 that contains main effect and interaction effect parameters of item *i*, and ***h***(**α**_*c*_, ***q***_*i*_) is a vector of the same length with linear combinations of the **α**_*c*_ and ***q***_*i*_. Particularly, $\lambda_{i}^{T}h\left(\alpha_{c},q_{i}\right)$ can be expanded to:

(5) $$\lambda_{i}^{T}h\left(\alpha_{c},q_{i}\right)=\sum_{a=1}^{A}\lambda_{i,1,(a)}\alpha_{ca}q_{ia}+\sum_{a=1}^{A-1}\sum_{a^{\prime }\;>a}^{A}\lambda_{i,2,(a,a^{\prime })}\alpha_{ca}\alpha_{{ca}^{\prime }}q_{ia}q_{ia^{\prime }}+\ldots ,$$

where λ_*i*, 1, (*a*)_ and $\lambda_{i,2,\left(a,a^{\prime }\right)}$ are the main effect for attribute α_*a*_ and the two-way interaction effect for α_*a*_ and $\alpha_{a^{\prime }}$. Since elements of **α**_*c*_ and ***q***_*i*_ are binary, ***h***(**α**_*c*_ , ***q***_*i*_) contains binary elements, which indicate effects that are estimates of interest. For an item measuring *n* attributes, *n*-way interaction effects should be specified in ***h***(**α**_*c*_, ***q***_*i*_). Table 2 provides a sample of a measure with three attributes: the first item that measures one attribute only (i.e., α_1_) has two estimates, where the third item which is associated with all given attributes contains eight estimates.

**Table 2:** A 3-item sample of expressions of a log-linear cognitive diagnostic model.

**Item**	**α_1_**	**α_2_**	**α_3_**	**$Expanded\;\lambda_{i,0}+\lambda_{i}^{T}h\left(\alpha_{c},q_{i}\right)\;expression$**	**Shortened expression**
1	1	0	0	λ_1,0_+λ_1,1_(1)+λ_1,2_(0)+λ_1,3_(0)+λ_1,12_(1 × 0)+λ_1,13_(1 × 0)+λ_1,23_(0 × 0)+λ_1,123_(1 × 0 × 0)	λ_1,0_+λ_1,1_(1)
2	0	1	1	λ_2,0_+λ_2,1_(0)+λ_2,2_(1)+λ_2,3_(1)+λ_2,12_(0 × 1)+λ_2,13_(0 × 1)+λ_2,23_(1 × 1)+λ_2,123_(0 × 1 × 1)	λ_2,0_+λ_2,2_(1)+λ_2,3_(1)+λ_2,23_(1)
3	1	1	1	λ_3,0_+λ_3,1_(1)+λ_3,2_(1)+λ_3,3_(1)+λ_3,12_(1 × 1)+λ_3,13_(1 × 1)+λ_3,23_(1 × 1)+λ_3,123_(1 × 1 × 1)	λ_3,0_+λ_3,1_(1)+λ_3,2_(1)+λ_3,3_(1)+λ_3,12_(1)+λ_3,13_(1)+λ_3,23_(1)+λ_3,123_(1)

## LCDM estimation

Traditionally, estimating LCDMs refers to the expectation maximization (EM) algorithm (Bock and Aitkin, 1981) that maximizes the marginal likelihood; this is the most commonly-seen algorithm in the CDM literature. In addition to the EM algorithm, Markov chain Monte Carlo (MCMC) techniques can be, theoretically, used to estimate the LCDM, but to date its application remains upon simpler CDMs such as the DINA model (da Silva et al., 2017; Jiang and Carter, 2018a). This study focuses on the EM algorithm due to its practicality and popularity. The EM algorithm is an intertwined updating mechanism consisting of E- and M-steps. With the provisional item parameter and probability of membership estimates from iteration *t*-1 (i.e., **λ**s and ***v***s), the posterior class probability for person *p* can be obtained in the E-step by

(6) $$H\left(C=c|Y_{p}=y_{p}\right)=\frac{v_{c}\prod_{i=1}^{I}\pi_{pi}^{y_{pi}}{\left(1-\pi_{pi}\right)}^{1-y_{pi}}}{\sum_{c=1}^{C}v_{c}\prod_{i=1}^{I}\pi_{pi}^{y_{pi}}{\left(1-\pi_{pi}\right)}^{1-y_{pi}}}$$

Based on Equation (6), the expected number of persons in latent class *c* and the expected number of persons in latent class *c* who answer item *i* correctly can be obtained by:

$$\begin{array}{l} n_{c}=\sum_{p=1}^{N}H\left(C=c|Y_{p}=y_{p}\right),\;and\\ r_{ci}=\sum_{p=1}^{N}y_{pi}H\left(C=c|Y_{p}=y_{p}\right), \end{array}$$

respectively. In the M-step, the following function is maximized with respect to item parameters **λ**:

$$\ell =\sum_{i=1}^{I}\sum_{c=1}^{2^{A}}\left[r_{ci}\log\pi_{ci}+(n_{c}-r_{ci})\log(1-\pi_{ci})\right],$$

and the probability of membership is updated by

$$\nu_{c}=\frac{\sum_{p=1}^{N}H\left(C=c|Y_{p}=y_{p}\right)}{N}.$$

Maximizing objective function ℓ usually requires Newton or Fisher scoring methods, where first- and second-order derivatives i.e., $\frac{\partial L}{\partial \lambda }\cdot {\left(\frac{\partial^{2}L}{\partial \lambda^{2}}\right)}^{-1}$ where the first component is a vector and the second component is a matrix) of the objective function are needed. If $\frac{\partial^{2}L}{\partial \lambda^{2}}$ becomes 0, the iteration process will stop and therefore fail to converge.

As a restricted latent class models, LCDM estimation faces the risk of local maxima (Jin et al., 2016). Theoretically, to obtain valid and accurate estimates, the model estimation should converge at a global maximum of the likelihood function, however, the mixture component of a mixture model is likely to trap the aforementioned EM updates to local maxima. In addition, label switching can occur and therefore lead to a misinterpretation of an estimation. For instance, a person mastering all attributes of interest can be mistakenly labeled as one with zero-mastery. Basing on the traditional EM approach, Rupp et al. (2010) add constraints to the parameter estimates (e.g., ensuring main effects are non-negative); this constraint approach substantially reduces the risks of local maxima and label switching (Lao and Templin, 2016). Using *Mplus* (Muthén and Muthén, 2013), a commercial software designed for latent variable modeling that by default deploys the traditional EM approach, Templin and Hoffman (2013) outline the procedures to specify syntax with parameter constraints for the LCDM estimation. Note that in the LCDM estimation, the EM approach in *Mplus* is turned into an accelerated version, meaning its updating steps are replaced with Quasi-Newton and Fisher scoring, this, however, still falls under the family of the traditional EM algorithm. Although Templin and Hoffman's *Mplus* practice has been implemented in many published works and is proved to be efficient (see Bradshaw and Templin, 2014; Li et al., 2016; Ravand, 2016 for example), it is still not avoiding the convergence failure issue: Templin and Bradshaw (2014) conduct a simulation study with vast conditions each of which was replicated 500 times, where the result shows the numbers of converged replications range from 330 to 447. To avoid the convergence issue while maintaining the properties of the EM approach, we introduce a machine-learning technique named Differential Evolution to estimate LCDMs.

## Differential evolution

**Figure 1:**
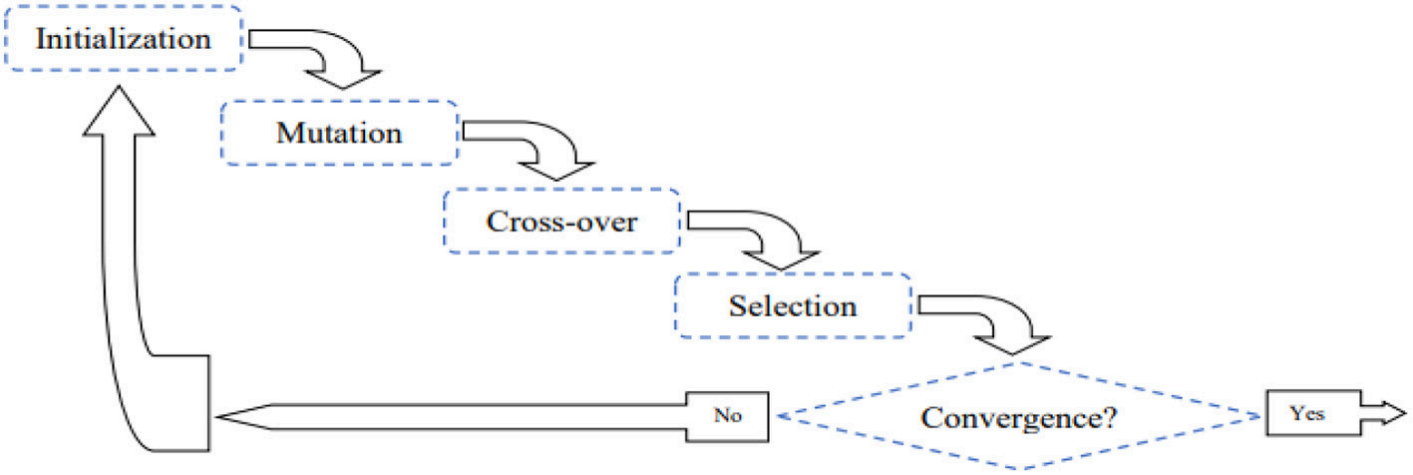
The iterative cycle of differential evolution algorithm.

Global optimization under machine-learning umbrella has gained tremendous attention from researchers, mathematicians as well as professionals in the field of engineering, finance, and scientific areas (Mohamed et al., 2012). Many applications of this kind impose complex optimization problems such traditional estimation techniques based upon derivatives become cumbersome or even impossible. To avoid the mathematical deriving procedures yet provide reliable solutions to complex models, Differential Evolution (DE) is invented (Storn and Price, 1997), developed, and applied to practice in different fields (e.g., Paterlini and Krink, 2006; Das et al., 2008; Rocca et al., 2011). Inspired by Darwinian evolution that entails the idea of mutation, crossover, and selection, DE is an enhanced version of derivative-free evolutionary algorithms and has been recognized as a simple yet efficient optimization approach in solving a variety of benchmark problems. The complete DE algorithm cycle can be found in Figure 1; in particular, the algorithm starts by sampling *D* candidate solutions to the problem of interest, where each candidate solution can be either a scalar or a vector (if there are more than one estimate). The mutation procedure takes place by performing simple arithmetic operations (i.e., addition, subtraction, and multiplication) among the existing solutions (namely parent solutions). The resultant mutation outcomes are then crossed over with the parent solutions to produce new candidate (offspring) solutions. Finally, in a one-to-one selection process of each pair of offspring and parent vectors, candidate solutions that fit the model better are passes into the next evolutionary cycle. This cycle iterates until the estimation converge. Mathematical and algorithmic details can be found in the following paragraph.

Let *R* be the number of estimates, **λ**_*LO*_ and **λ**_*HI*_ be the lower and the upper limits (vectors) of the parameters of the estimates, and *G*(·) be the objective function. Initial candidate solutions **λ**_*d*_ = (λ_*d*1_, λ_*d*2_, …, λ_*dR*_) for *d* = 1, 2,…, *D* can be generated by (1) randomly drawing samples from certain distribution(s) or (2) specifying values with educated guesses, where *D* is the number of candidate solutions. Mutation procedure can be achieved via different strategies: (1) DE /rand/ 1, (2) DE/current-to-rest/1, and (3) DE/best/1. In particular, for a given set of candidate solutions **λ**_*d*_ for *d* = 1, 2,…, *D*, the mutation outcomes ***m***_*d*_ can be calculated as:

$$\begin{array}{cc} DE/rand/1 & m_{d}=\lambda_{\delta_{o}}+F_{d}*(\lambda_{\delta_{1}}-\lambda_{\delta_{2}})\\ DE/current-to-rest/1 & \begin{array}{l} m_{d}=\lambda_{\delta_{o}}+F_{d}*(\lambda_{best}-\lambda_{\delta_{2}})\\ \;+F_{d}*(\lambda_{\delta_{1}}-\lambda_{\delta_{2}}) \end{array}\\ DE/best/1 & m_{d}=\lambda_{best}+F_{d}*(\lambda_{\delta_{1}}-\lambda_{\delta_{2}}) \end{array}$$

Where δ_*o*_, δ_1_, and δ_2_ are distinct integers uniformly sampled from 1 to *D*, **λ**_δ_1__ − **λ**_δ_2__is the difference vector that would be used to mutate two selected parent candidates (e.g., DE/rand/1), **λ**_*best*_ is the best candidate solution at the current iteration, and finally *F*_*d*_ is the mutation scaling factor that is randomly drawn from a uniform distribution on the interval (0, 1). Some ***m***_*d*_ may be produced beyond the constraints set by **λ**_*lo*_ and **λ**_*hi*_; some effective solutions to the violation include (1) re-generating a candidate solution until it is valid and (2) setting penalty to the objective function. If an element r in a candidate solution encounter the boundary issue, a quick fix by setting the violating elements to be the middle between boundaries and the that of its parent solution. That is, $m_{dr}=\frac{\lambda_{LOr}+\lambda_{dr}}{2}$ for *m*_*dr*_ < **λ**_*LOr*_ and $m_{dr}=\frac{\lambda_{HIr}+\lambda_{dr}}{2}$ for *m*_*dr*_ > **λ**_*HIr*_. After obtaining ***m***_*d*_ from the mutation procedure, a “binomial” crossover operation forms the offspring candidate solutions: let *CR* be a crossover probability that controls the fraction of the elements that are copied from the parent candidate solution and *u*_*dr*_ be a candidate solution, if a random number *z*_*r*_ sampled from a uniform distribution (0, 1) is smaller than *CR*, the element *r* of the offspring of *u*_*dr*_ is *m*_*dr*_, and λ_*dr*_ otherwise. The default *CR* is usually set to 0.5 for a balanced stochastic move. Finally, if *G*(***u***_***d***_) is better than *G*(**λ**_***d***_), ***u***_***d***_ would replace **λ**_***d***_ to serve as a parent solution for the next iteration. The DE algorithm can be tailored to a parallel computing platform; technically each candidate solution can be calculated in an independent computational unit such that queuing time can be shortened. That said, instead of sequentially updating the candidate solutions, a parallel DE algorithm can perform simultaneous updates.

To illustrate how the DE algorithm functions, an example of a simple regression estimation is provided here. Let independent variable ***x*** = [22, 14, 15, 12, 10, 26, 11, 28] and dependent variable ***y*** = [44, 29, 30, 27, 24, 51, 25, 56] resulting in $\hat{\beta }$ = [4.98, 1.78] with the ordinary least squares (OLS) estimator. Using the DE algorithm in this case sets the objective function *G*(λ) to $-1^{*}\sum {\left(y-\hat{y}\right)}^{2}$, which ideally should be maximized to −7.19 according to the OLS result. To keep the demonstration simple, let the number of candidate solutions *D* = 3 and initial values for **λ**_1_, **λ**_2_, and **λ**_3_ were arbitrarily set to [2, 1], [−3, 5], and [1, 2]. At the initial iteration, the best solution was [1, 2] as *G*(**λ**_**3**_) = −24 where *G*(**λ**_**1**_) and *G*(**λ**_**2**_) were −2400 and −21672. Therefore, **λ**_*best*_ at this stage became **λ**_**3**_. With certain random draws for a given mutation calculation (e.g., DE/best/1), ***m***_1_, ***m***_2_, and ***m***_3_ happened to be [3.5, 0.8], [−1, 3.5], and [2, 1.8]. Let *CR* = 0.5, if a random generation produced *z*_1_ = 0.7 and *z*_2_ = 0.4 for example, the first offspring ***u***_**1**_became [3.5, 1] by taking elements from **λ**_**1**_ and ***m***_1_. This resulted in *G*(***u***_**1**_) = −2022, which is larger than *G*(**λ**_**1**_), and therefore the new **λ**_**1**_ would be replaced by ***u***_**1**_. On the other hand, if ***u***_**3**_ became [1, 1.8] which produced *G*(***u***_**1**_) = −116.8, then the **λ**_**3**_ remained still. This process continues until *G*(**λ**) converges to −7.19.

In this paper, we integrated the DE into the EM algorithm to estimate LCDMs^1^ To make the proposed approach easy to follow, we name it EM-DEoptim algorithm from here. Especially, the method for updating item parameters within the M-step is replaced by the DE algorithm, while the rest of the EM procedures remain identical. To be concrete, the objective function that the EM-DEoptim maximizes is Equation 3, given *v*_*c*_ for each latent class is known. As the DE is a stochastic and global optimization technique, the EM-DEoptim is expected to encounter fewer occurrences of the local maxima problem than the traditional EM algorithm (Celeux et al., 1996). In addition, as addressed above, the EM-DEoptim is based upon derivative-free framework such that it can be easily fitted to arbitrarily customized LCDMs without re-deriving the gradient functions nor re-approximating information matrix. For example, if constraining the main effects of Item *i* and Item *i'* to be equal while still allowing others to be estimated freely is needed, the EM-DEoptim algorithm can handle the situation by simply assigning the same labels to the constrained parts in the likelihood function expression, where the traditional EM algorithm needs altering the derivatives. This advantage can effectively prevent the aforementioned un-differentiable situations. Last but not least, the computational speed of the EM-DEoptim algorithm, although not outperform the traditional EM algorithm in a singular operation environment, can be substantially improved via parallel computing facilities that are naturally suited to modern machine-learning-based techniques.

## Simulation study

We conducted a simulation study to demonstrate the utility of the EM-DEoptim algorithm. Specifically, the study involved two investigations: the number of times that the traditional EM algorithm fails and the comparison between the EM-DEoptim algorithm and the traditional EM algorithm in terms of the parameter recovery. In the simulation study, the numbers of attributes *A* were set to 3, 4, 5. The Q-matrix was randomly generated: when there were 3 attributes (*A* = 3), a balanced Q-matrix in which each item measures either one or two attributes was utilized; similarly, at the condition of 4 and 5 attributes, each item measures two to three attributes. The number of items *I* was set to 30 and the number of persons *N* was set to 300. The attributes were generated via two steps: continuous values were initially generated from a multinormal distribution *MV* (**0**, Σ) of which the diagonal elements of Σ were constrained to 1 and the off-diagonal values (i.e., correlations between attributes) were randomly drew from a uniform distribution ranging from 0.7 to 0.9, and these continuous values were further converted onto the binary scale by comparing the values with zero (i.e., 1 if the value is larger than zero and 0 otherwise). Finally, the item parameters were specified to two level: high-quality group that sets main effects = 2, intercepts = −1.5, and interaction effects = 0.5, and low-quality group that makes main effects = 0.2, intercepts = −0.5, and interaction effects = 0.1.

The traditional EM algorithm was realized via the package *CDM* (George et al., 2016; alternatively, one can choose the package *GDINA* by Ma and de la Torre, 2018), where the EM-DEoptim algorithm was executed in *R* (R Core Team, 2018). The stop criterion in *CDM* was set to 1,000 iterations or the change of likelihood value < 0.001, where the EM-DEoptim algorithm was forced to stop if the iteration number reaches to 1,000 or the likelihood value remains identical for 10 iterations. In this study, the DE configurations were set to default (Ardia et al., 2011): DE/current-to-rest/1 with *F*_*d*_ = 0.8, *CR* = 0.5, and 500 candidate solutions, where ± 20 is used to constrain the parameter estimates. The machine used was Dell Precision 3520 with 16GB RAM and a 2.90 GHz i7-7820 4-core Intel processor. The study was replicated for 200 times.

The dependent variables in this part of the study are (1) the number of convergence failure of the EM algorithm, (2) relative bias (RBIAS) and root mean squared error (RMSE), and (3) the attribute classification accuracy measured by each attribute and each profile. Overall, there was only two failed convergence failures when the item quality was high, where the low-quality item parameters led to seven failures: two cases in the situation of *A* = 4 and five cases when *A* = 5. On the other hand, EM-DEoptim had no unexpected terminations during the iterations. Table 3 shows the attribute classification accuracy rates. Both algorithms produced very similar results, where some patterns can be discovered: (1) the more attributes the estimation face, the less accurate the attribute estimates are yielded, (2) the higher the item parameter quality is, the more accurate the attribute estimates are produced, and (3) the profile accuracy is more sensitive to the item parameter quality.

**Table 3:** attribute accuracy rate of the simulation study.

		**EM**		**DE-EMoptim**	
***A***	**Quality**	**Attribute**	**Profile**	**Attribute**	**Profile**
3	High	0.848	0.634	0.847	0.642
4	High	0.816	0.505	0.821	0.489
5	High	0.768	0.336	0.755	0.342
3	Low	0.516	0.104	0.517	0.104
4	Low	0.509	0.039	0.513	0.044
5	Low	0.504	0.017	0.468	0.013

Similar to the attribute estimates, the item parameter recovery presented similar pattern for both algorithms as listed in Table 4. The biases and MSEs were higher when (1) the number of attributes was larger and (2) the item parameter quality is higher. In addition, main effect estimates were more accurate and efficient than both interaction and intercept effects. This finding is not uncommon in complex psychometric models (Jiang et al., 2016). When the item parameter quality is low, and/or the number of attribute is large (e.g., 5), the EM-DEoptim performed better than the traditional EM algorithm. An important reason is that the boundary constraints imposed by the EM-DEoptim algorithm can limit the estimates into a certain range. Although not a main focus of the studies, the computing speed showed a substantial difference: the average time (in seconds) for 3-, 4-, and 5 attributes were 4.45, 22.55, and 78.64 for the traditional EM algorithm, while the EM-DEoptim took 61.22, 354.18, and 1228.76.

**Table 4:** Item parameter estimates of the simulation study.

**RBIAS**		**EM**			**EM-DEoptim**		
***A***	**Quality**	**Main**	**Intercept**	**Interaction**	**Main**	**Intercept**	**Interaction**
3	High	−0.433	−0.270	−1.896	−0.463	−0.280	−1.196
4	High	−1.744	−0.948	−5.036	−1.740	−0.938	−4.106
5	High	−1.906	−0.957	−4.975	−1.906	−0.957	−2.675
3	Low	−5.389	−8.906	−2.160	−2.389	−4.406	−1.960
4	Low	−9.526	−9.950	−3.696	−8.626	−7.950	−4.026
5	Low	−11.99	−6.419	−10.027	−9.495	−5.419	−7.027
**RMSE**		**EM**			**EM-Deoptim**		
3	High	6.346	2.124	8.349	6.890	1.924	4.336
4	High	14.576	5.035	15.679	15.079	6.422	13.853
5	High	21.643	6.230	26.327	18.223	7.360	18.707
3	Low	15.423	11.630	18.478	12.863	12.112	18.481
4	Low	22.512	15.165	26.064	19.299	16.865	17.446
5	Low	25.410	15.109	15.319	16.506	14.409	14.319

## Real data application

**Figure 2:**
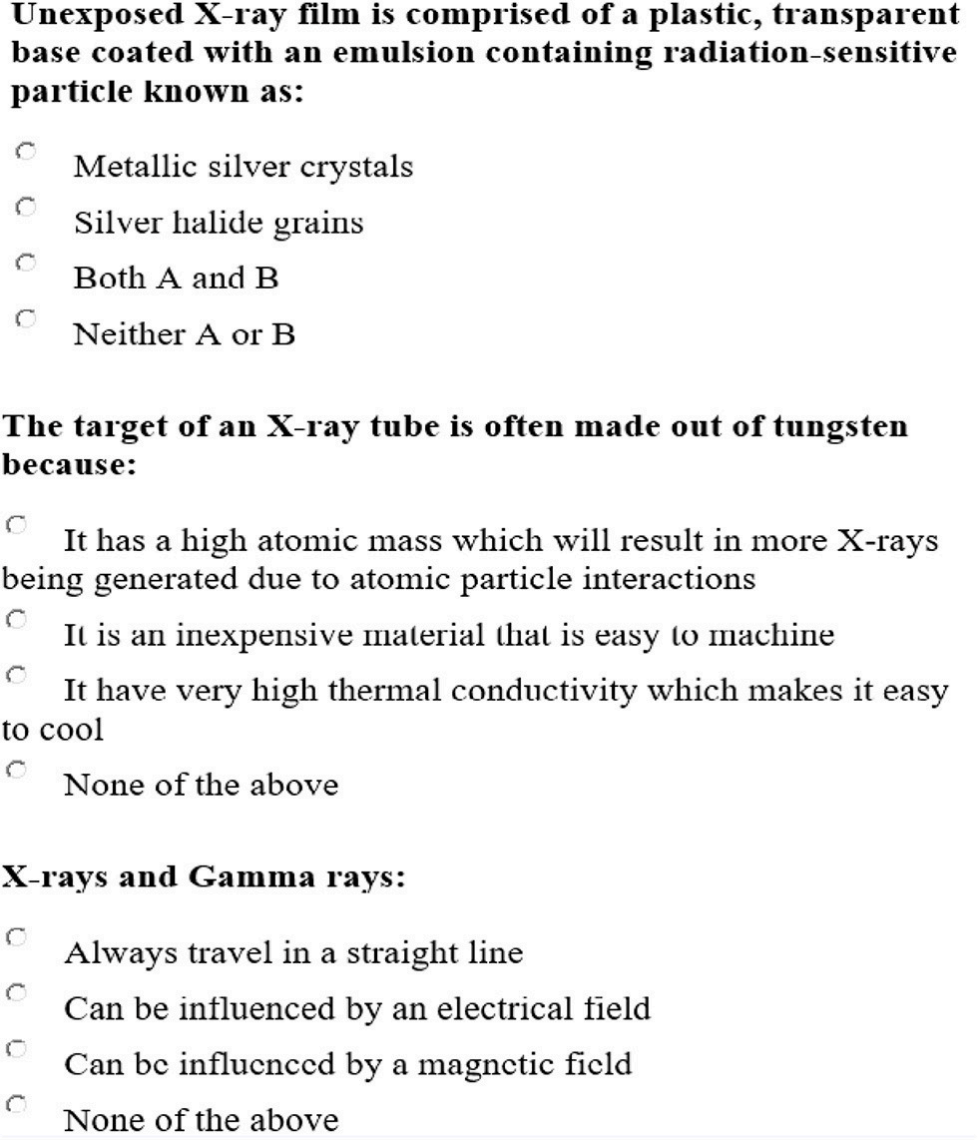
Three sample items of the health profession test.

The dataset used in this session is an assessment of a health profession administered to 3491 test takers (Jiang and Raymond, 2018). The number of items is 200 each of which measures one attribute. Therefore, there are five attributes in total: the knowledge of radiation biology (Items #1-45), the knowledge of equipment operation (Items #46-67), the image acquisition and evaluation capacity (Items #68-112), the knowledge of imaging procedures (Items #113-162), and ethics (Items #163-200). Three samples of the items can be found in Figure 2.

Two common model fit indices are reported as: (1): mean of absolute deviations in observed and expected correlations (MADcor; DiBello et al., 2007) is 0.041 and standardized mean square root of squared residuals (SRMSR; Maydeu-Olivares, 2013; Maydeu-Olivares and Joe, 2014) is 0.05. Overall, the model has an adequate fit. Note that more model fit indices such as χ^2^-like statistics (Orlando and Thissen, 2000) are recommended. This paper focuses on the estimation. More model fit details can be found in Hu et al. (2016) and Sorrel et al. (2017).

**Figure 3:**
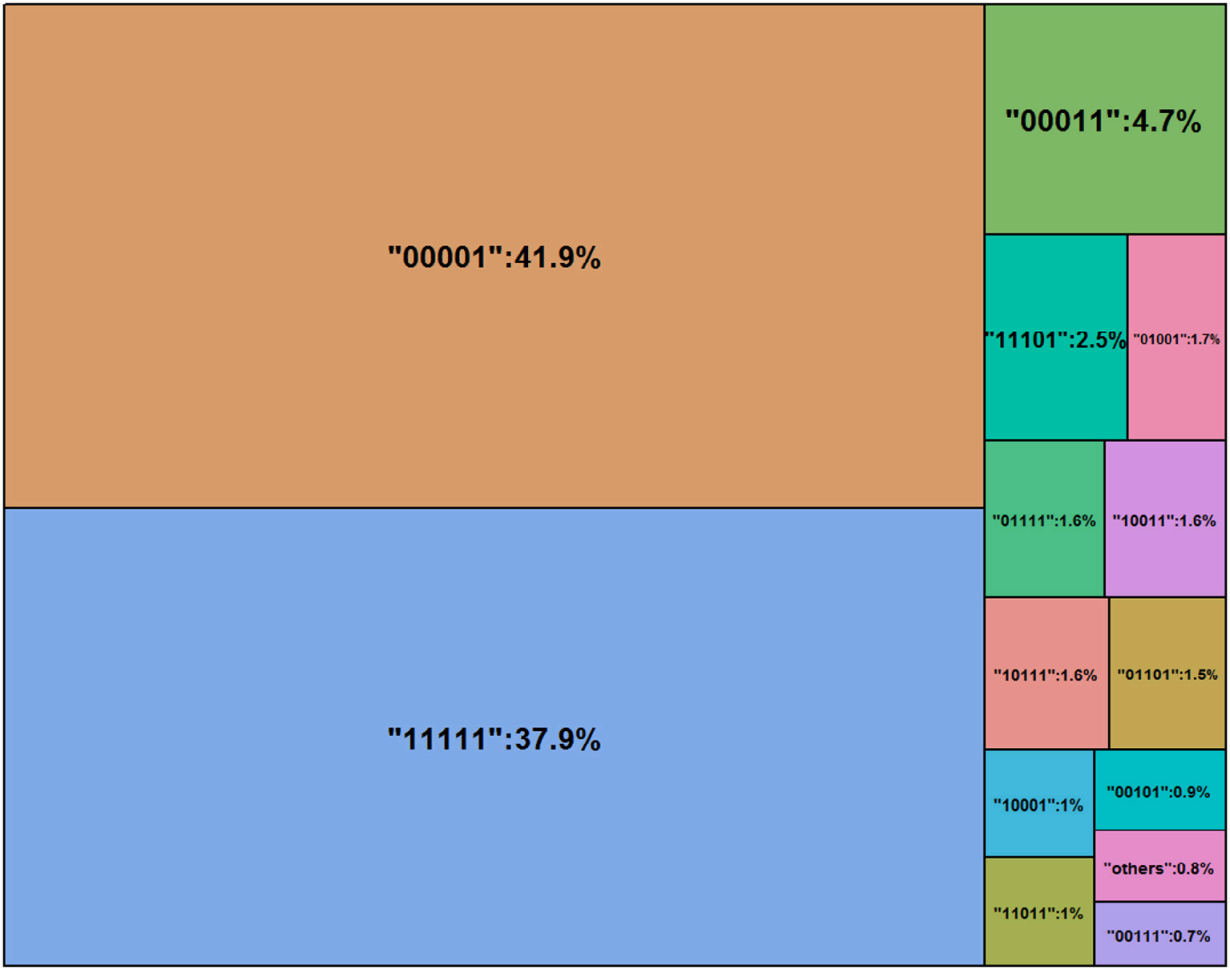
Estimated class probabilities via LCDM.

**Figure 4:**
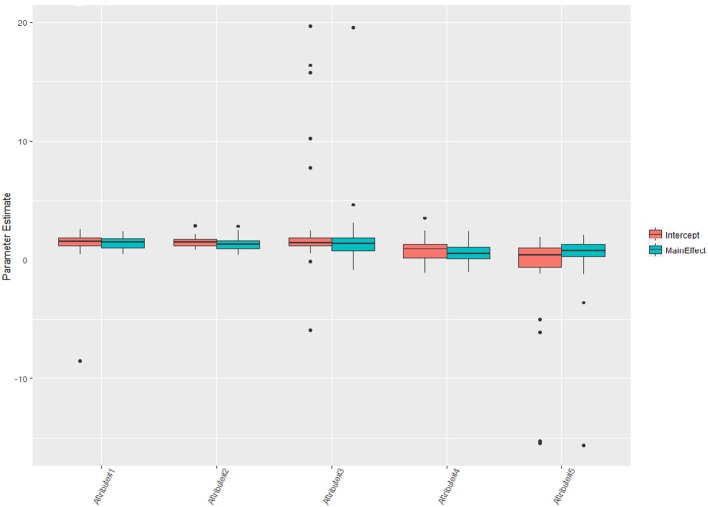
Item parameter estimates of the health profession test.

Rounding the number of digits to three after the decimal point, one can see that 16 classes are nearly empty and therefore are labeled as “others” in Figure 3 (see Jiang and Carter, 2018b for more visual aids). Nearly 40% of the test takers master all five attributes. According to Templin and Bradshaw (2014), many empty classes indicate potential hierarchies of attribute structure, however, the parameter estimates can be relatively robust even the non-hierarchical modeling is adopted here. Figure 4 shows the distributions of the parameter estimates grouped by parameter types and attribute identifications. Attribute #3 had the highest means of both intercepts and main effects: 2.65 and 1.79. The means of intercepts and main effects of Attribute #5 were −1.05 and 0.20.

To compare the estimates with other estimation approaches, we also implemented a Bayesian technique-Hamiltonian Monte Carlo-to the analysis by adopting uninformative priors for both item parameters and the class membership probability: the mean and standard deviation for item parameters were 0 and 20, while the Dirichelet prior parameters were all set to 1 (see Jiang and Carter, 2018a for details). The correlations of item parameter estimates were relatively high: 0.77, 0.84, and 0.69 for intercept, main effect, and interaction effects. On the other hand, the attribute agreement was lower than that of the item parameter estimates: the average ratio for all attributes was 0.67, where the value dropped to 0.39 when it comes to the match of the class membership classification. This makes sense as the Dirichelet prior had forced the assignment on each latent class and therefore the result tended to be more different from those that were fully determined by the EM algorithm.

## Discussion and conclusion

The purpose of this paper is to propose a machine-learning based algorithm for the estimation of LCDMs. In particular, the proposed estimator is a combination of the EM framework and the DEoptim algorithm, which has been popular in neural networks and business analytics fields. The performance of the proposed algorithm is evaluated through a simulation study of which the results indicate that it is an appropriate option to handle LCDM estimation task. This paper, however, does not suggest that the proposed algorithm should replace the EM algorithm in practice; at the situations where the EM algorithm fails to produce estimates due to the unsuccessful derivative updates, the EM-DEoptim algorithm can be an alternative.

The proposed EM-DEoptim algorithm and the traditional EM algorithm implemented in M*plus* produced virtually identical parameter estimates, and the former seems less frequently to fail. The average computational time for M*plus* estimation with the multiple-core option is 15 min. The difference is caused by the features of the algorithms: the EM algorithm based upon Quasi-Newton and Fisher scoring updates estimates with directional steps (i.e., the iteration always leads to better solutions), while the DEoptim part is truly stochastic such that the updating procedures may be wasted. Even though the DEoptim mechanism is fundamentally less directional than Quasi-Newton and Fisher scoring, The EM-DEoptim algorithm perform cannot very similar to the EM algorithm. Theoretically, the EM-DEoptim algorithm can be many times faster than what it is now if the entire function is constructed in *C*++ or *Fortran*; currently only the DEoptim is implemented in *C*++ through the package *RcppDE*, where the entire algorithm is written in base *R* software scripting language. Research has shown that using compiler package with *R* often takes less than half of time executing the same function than that of without packages (e.g., Aruoba and Fernández-Villaverde, 2015). In addition, given the DEoptim algorithm is composed of basic calculation, performing the proposed algorithm in a vectorization approach and therefore with graphics processing units (GPUs) is expected to accelerate the estimations.

## Author contributions

ZJ proposes the idea about integrating differential evolution optimization into the EM framework in the LCDM estimation and deploys the functional algorithm in simulation studies; WM produces both literature review and technical detail supports to this manuscript.

### Conflict of interest statement

The authors declare that the research was conducted in the absence of any commercial or financial relationships that could be construed as a potential conflict of interest.

## Footnotes

^1^The snippet code can be found https://alabama.box.com/s/cbuxetk19b1pk1invi1gnbqxd8hw5fij.
